# Postgraduates’ time management disposition and mental health: mediating role of life satisfaction and moderating role of core self-evaluations

**DOI:** 10.1186/s40359-023-01349-2

**Published:** 2023-10-06

**Authors:** Shen Liu, Minghua Song, Han Teng

**Affiliations:** 1 Department of Psychology, School of Humanities and Social Sciences, Anhui Agricultural University, Hefei, 230036 Anhui China; 2 Mental Health Education Guidance Center, Huzhou University, Huzhou, 313000 Zhejiang China

**Keywords:** Time management disposition, Life satisfaction, Core self-evaluations, Mental health, Postgraduates

## Abstract

**Background:**

The current study aimed to investigate the relationship between postgraduates’ time management disposition and mental health. As such, it constructed a moderated mediation model to examine the mediating role of life satisfaction on the relationship between graduate students’ time management disposition and mental health and examine whether this role was moderated by core self-evaluations.

**Methods:**

455 postgraduates were surveyed by the Adolescence Time Management Disposition Inventory, the Adolescent Students’ Life Satisfaction Scale, the revised version of the Chinese Core Self-Evaluation Scale, and the revised version of the Chinese General Health Questionnaire.

**Results:**

Time management disposition, life satisfaction, core self-evaluation, and mental health were significantly correlated. Time management disposition indirectly affected mental health through the mediating effect of life satisfaction. Core self-evaluation moderated the second half of the mediating effect of time management disposition on mental health via life satisfaction.

**Conclusion:**

The findings reveal the mechanism between time management disposition and mental health, which will help school educators to guide postgraduates in developing good time management disposition and improving life satisfaction and core self-evaluation, and thus improve their mental health.

## Introduction

With the rapid development of society, transformation of educational strategies, and continuous expansion of postgraduate enrollment, postgraduate groups are faced with multiple pressures from their studies, families, and society and are prone to mental health problems such as anxiety and depression [1]. The mental health of postgraduates, as a higher-level group in the education system, is not only related to their own academic progress and healthy growth, but it also has an important impact on the development of an entire country’s scientific research level. Therefore, it is necessary to mind and improve the mental health of postgraduate students and improve their quality of life [1]. However, most studies on the influencing factors of graduate students’ mental health have constructed an inhibitory model of “negative traits (e.g., depression, anxiety, aggression) → positive factors (e.g., achievement motivation, self-efficacy, empathy)”, and explored the influencing factors, such as the failure of social media self-control [2, 3]. However, the development model of “positive traits (e.g., confidence, self-esteem, optimism) → positive factors (e.g., achievement motivation, self-efficacy, empathy)” has rarely been constructed to improve postgraduates’ mental health level. Therefore, the current study attempts to clarify the internal mechanism of the positive factors affecting postgraduates’ mental health and thus provide a reference for formulating relevant measures to improve postgraduates’ adaptability and maintain their mental health.

The academic community highly ranks time management disposition among the many factors that affect the mental health of graduate students [4, 5]. Time management disposition refers to individuals’ psychological and behavioral characteristics regarding how they use time based on their understanding of the value and meaning of time [2, 6]. It is not only the cognitive characteristics of an individual’s attitude, planning, and utilization of time but also an individual’s values and behavioral tendencies towards time. Further, it is a personality characteristic with a multidimensional and multilevel psychological structure [7]. According to the process model of time management, time planning and monitoring can improve the judgment ability of self-time management and thus increase positive experiences [8]. For example, individuals who managing time well have a positive sense of self-reliance, self-concept, and self-confidence; are more likely to realize self-worth; and tend to have higher levels of mental health [9]. However, individuals who neglect time management have a relatively low level of self-confidence and are prone to a strong sense of inferiority, increased anxiety, and low self-evaluation [8]. It is easy to identify a close relationship between time management disposition and mental health.

Life satisfaction refers to individuals’ overall cognitive assessment of their life status for some or most of the time, according to the standards they construct [10]. As a cognitive component, life satisfaction is a key factor and an important indicator for measuring mental health [11]; it is also an essential feature and the core of mental health [12]. In their longitudinal study, Fergusson et al. found a strong correlation between life satisfaction and mental health [13]. Previous studies have found that improving individuals’ life satisfaction can help promote their academic development, while preventing them from developing psychological and behavioral problems [14, 15]. Individuals with lower life satisfaction may adopt negative coping styles and experience more negative emotions (such as depression), which would seriously affect their mental health [16, 17]. In addition, time management disposition positively predicts life satisfaction [18, 19]. Specifically, individuals who manage and use their time well are more likely to remain calm and be at ease in life and in their studies, be praised by their teachers and classmates, and experience increased positive emotions such as happiness and satisfaction, thereby improving their self-esteem, self-confidence levels, and life satisfaction [9]. In addition, individuals with a good time management disposition will use their time more rationally, complete study and work tasks efficiently, and free up more time to develop hobbies and participate in recreational activities. This not only helps to improve their life satisfaction but also helps to relieve academic pressure, thereby reducing the risk of psychological problems [20]. Therefore, the current study proposes hypothesis *H*_1_: Life satisfaction mediates the relationship between time management disposition and mental health.

Core self-evaluation is the most basic evaluation and estimation of individuals’ self-capacity and value, and it is a deep-level personality resource structure [21]. According to the benefit mechanism of self-evaluation, individuals with high self-evaluation can perceive more positive cognitive emotions, thereby maintaining their positive emotional experiences [22]. The study found that core self-evaluation not only has an important positive significance on individuals’ mental health, well-being, and life satisfaction, but it can also moderate the negative effects of adverse environmental stimuli [23, 24]. At the same time, according to the protective-protective model, different protective factors may interact when predicting developmental outcomes; that is, the predictive effect of a protective factor (such as time management tendency) on the outcome variable (mental health) may vary depending on the level of another protective factor, such as core self-evaluation [25]. It is speculated that time management disposition and core self-evaluation may jointly affect mental health. Compared with individuals with low core self-evaluation, individuals with high core self-evaluation levels have a stronger sense of control in their daily life and studies, believe in their own abilities, and recognize their own values; thus, they effectively inhibiting the negative impact of anxiety and depression [25]. In addition, individuals with high core self-evaluation are better at time management and reduce the stress caused by the lack of time [26], thus reducing the possibility of psychological problems. As such, the current study proposes hypothesis *H*_2*a*_: Core self-evaluation moderates the relationship between time management disposition and mental health.

As a positive psychological quality, core self-evaluation not only positively affects life satisfaction [27], but also the prediction of life satisfaction on mental health [28]. Research shows that individuals with high core self-evaluations tend to set more aggressive goals; access more resources to deal with challenges caused by life and work conflicts; demonstrate strong time management skills [29]; and are good at choice and self-harmony interests, values, and growth needs, thereby increasing job and life satisfaction [27]. At the same time, according to the promotion hypothesis of the protection factor-protection factor model [30] and the time management process model [31], core self-evaluation will amplify or enhance the positive effect of time management disposition on life satisfaction. In other words, when core self-evaluation is high, individuals with high time management disposition are more efficient and more likely to meet their own needs, thereby improving their life satisfaction level. When core self-evaluation is low, individuals with high time management disposition still have higher life satisfaction compared to those with low time management disposition; however, important psychological resources are reduced and the predictive effect of time management tendency on life satisfaction may be relatively weak. Based on this, the current study proposes hypothesis *H*_2*b*_: Core self-evaluation moderates the relationship between time management disposition and life satisfaction.

In addition, an increase in individual life satisfaction, if sustained, will promote more positive outcomes [32], thereby reducing the risk of psychological problems. According to conservation of resources theory, core self-evaluation may enhance the positive effect of life satisfaction on mental health [33]. Specifically, individuals with high core self-evaluation are better at coping with negative emotions [34] and less likely to have psychological problems [35]. In contrast, individuals with low core self-evaluations are more prone to psychological problems, such as anxiety and depression [36]. On this basis, the current study hypothesizes that *H*_2*c*_: Core self-evaluation moderates the relationship between life satisfaction and mental health.

**Fig. 1:**
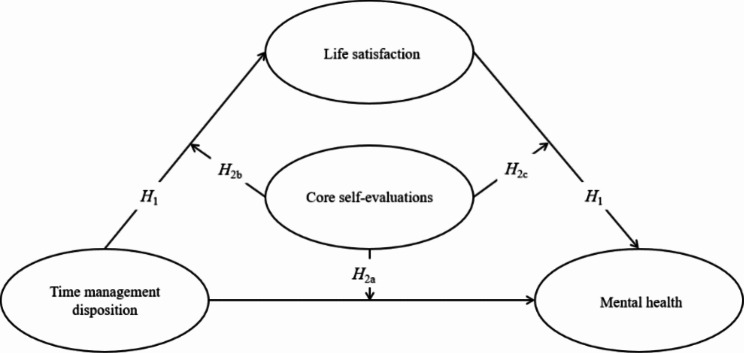
Theoretical model

In conclusion, the current study constructed the theoretical model shown in Fig. 1 to explore the role of life satisfaction and core self-evaluation in the effect of time management disposition on mental health. We proposed these hypotheses including life satisfaction mediates the relationship between time management disposition and mental health (*H*_1_), core self-evaluation moderates the relationship between time management disposition and mental health (*H*_2*a*_), core self-evaluation moderates the relationship between time management disposition and life satisfaction (*H*_2*b*_), and core self-evaluation moderates the relationship between life satisfaction and mental health (*H*_2*c*_).

## Methods

### Study design and participants

An a priori power analysis revealed that to test a simple mediation model with an anticipated small effect size for the view between time-management disposition to life satisfaction and between life satisfaction to mental health indicated a minimum of approximately 450 participants would be needed to test for mediation using the parametric bootstrap procedure for calculating the standard error of the indirect effect [37]. In order to adequately power a test of moderated mediation, we anticipated needing a minimum sample size of 400 participants (using bootstrapped procedure to estimate SE) for potential moderated mediation given a small effect size [38]. We selected students from three universities in Anhui and Hubei, China. We chose these three universities because they presented rural, industrial, and economic territories. 480 postgraduates were recruited via questionnaires and scales. Among them, 25 postgraduates were excluded owing to missing or consecutive answers the same option. Finally, 455 valid data points were obtained with an effective recovery rate of 94.79%. Among the 455 participants, 329 were male and 126 were female between 23 and 30 years old, with an average age of 23.31 years (*SD* = 1.72). In addition, there were 397 postgraduates in the first grade, 25 postgraduates in the second grade, and 33 postgraduates in the third grade and above. All subjects participated in the study voluntarily. After completing the study, the subjects received certain rewards (20 RMB) or equivalent gifts (a pen or a notebook). The Ethics Committee of Anhui Agricultural University approved the current study, in accordance with the ethical principles of the Declaration of Helsinki. All subjects gave written informed consent in accordance with the ethical principles of the Declaration of Helsinki.

### Measures

#### The adolescence time management disposition inventory

Time management disposition was measured using the Adolescence Time Management Disposition Inventory [6]. The inventory includes three dimensions with 44 items: time value (e.g., “no matter what I do, the first thing I have to consider is time”), time monitoring concept (e.g., “I usually organize my daily activities into a schedule”), and time efficacy (e.g., “I think my time allocation between study and extracurricular activities is reasonable”).The higher the average score, the higher the participant’s time management disposition, with 1 meaning “totally disagree” and 5 meaning “totally agree.” The subscales scores of the time value, time monitoring concept and time efficacy were respectively 35.20, 79.33, and 34.71. In the current study, Cronbach’s α coefficient of the inventory, time value, time monitoring concept and time efficacy were respectively 0.94, 0.81, 0.90, 0.78. In the current study, the second-order CFA model generated a very good fit with χ^2^/*df* = 2.50, RMSEA = 0.058, CFI = 0.95, NFI = 0.94, GFI = 0.90, and both the absolute and value-added adaptation indexes were within the acceptable range.

#### The adolescent students’ life satisfaction scale

Life satisfaction was measured using the Adolescent Students’ Life Satisfaction Scale [39]. The scale includes six dimensions with 36 items: friendship (e.g., “my friends respect me”), family (e.g., “I like to be with my parents”), study (e.g., “I have achieved desirable academic achievement”), freedom (e.g., “basically no one is forcing me to do things I don’t like to do”), school (e.g., “I feel uncomfortable at school”), and environment (e.g., “there are many unpleasant things around the environment I live in”). The higher the average score, the higher the participant’s life satisfaction, with 1 meaning “totally disagree” and 7 meaning “totally agree.” The subscales scores of the friendship, family, study, freedom, school, and environment were respectively 38.13, 39.00, 28.98, 27.49, 31.86, and 27.01. In the current study, Cronbach’s α coefficient of the scale, friendship, family, study, freedom, school, and environment were respectively 0.92, 0.79, 0.90, 0.87, 0.65, 0.85, 0.62. In the current study, the second-order CFA model generated a very good fit with χ^2^/*df* = 0.35, RMSEA = 0.020, CFI = 0.95, NFI = 0.94, GFI = 0.95, and both the absolute and value-added adaptation indexes were within the acceptable range.

#### The revised version of the Chinese core self-evaluation scale

Core self-evaluations were measured using the revised version of the Chinese Core Self-Evaluation Scale [40]. The scale has only one dimension with 10 items (e.g., “I believe in myself to be successful in life”). The higher the average score, the higher the participant’s core self-evaluations, with 1 meaning “totally disagree” and 5 meaning “totally agree.” In the current study, Cronbach’s α coefficient of the scale was 0.88. In the current study, the second-order CFA model generated a very good fit with χ^2^/*df* = 3.98, RMSEA = 0.061, CFI = 0.96, NFI = 0.94, GFI = 0.95, and both the absolute and value-added adaptation indexes were within the acceptable range.

#### The revised version of the Chinese general health questionnaire

Mental health was assessed using the revised version of the Chinese General Health Questionnaire [41]. The questionnaire includes three dimensions with 20 items: self-affirmation (e.g., “basically everything is happy”), depression (e.g., “I lose confidence in myself”) and anxiety (e.g., “I feel uneasy and nervous all day”). The higher the average score, the higher the participant’s mental state, with 1 meaning “yes” and 0 meaning “no”; depression and anxiety were scored in reverse. In the current study, Cronbach’s α coefficient of the questionnaire was 0.90. In the current study, the second-order CFA model generated a very good fit with χ^2^/*df* = 3.87, RMSEA = 0.004, CFI = 0.93, NFI = 0.95, GFI = 0.95, and both the absolute and value-added adaptation indexes were within the acceptable range.

### Data collection

After obtaining the informed consent of the students themselves, this study was conducted a collective survey on a class basis. The participants filled out the questionnaires in a quiet classroom, with trained psychology students as the subjects. Before conducting the survey, the subjects explained the requirements, the instructions, and provided timely guidance when the participant encountered problems. The participants anonymously filled out the questionnaires, collected it on the spot for numbering (approximately 30 min). After the end, the subjects explained the research purpose to the participants and asked if they have guessed the research purpose.

### Data analysis

Owing to common method biases, during the test process, participants were informed that the research questionnaires were limited to academic research, their personal information would be confidential, and their right to withdraw or suspend the research would be respected. Meanwhile, we conducted a common method bias test based on Harman’s single factor test, in which less than 40% indicated no significant common method bias [42]. SPSS 22.0 was used to provide demographic variables and descriptive statistics of the four studied variables and correlation matrices and thus further control the variables and perform model tests. Model 59 of the PROCESS macro program (download address: http://www.Afhayes.com/) written by Hayes was used to perform the moderated mediating model test [43].

## Results

### Common method biases test

All data were collected from the participants’ self-reports; therefore, the results were susceptible to common method bias [42]. Following Zhou and Long, Harman’s single-factor test was adopted as the common method bias test [44]. The results showed that 24 components had eigenvalues greater than 1.0, and the largest single component accounted for 18.13% of the variance, far less than 40%. This indicates that the current study’s results had no obvious common method bias.

### Descriptive analysis

Table 1 shows the mean, standard deviation, and correlation matrix of the variables used in the current study. The results showed that time management disposition, life satisfaction, and self-evaluation were significantly positively correlated with mental health; time management disposition, life satisfaction, and self-evaluation were significantly positively correlated; and time management disposition and life satisfaction were significantly positively correlated. This suggests that the data obtained in the current study can be used for further analysis.

**Table 1:** Means, standard deviations, and correlation matrices of variables (*n* = 455)

	*M*	*SD*	1	2	3	4	5	6	7
1. Gender	—	—	—						
2. Age	23.31	1.72	0.07	—					
3. Grade	—	—	-0.06	0.44^**^	—				
4. Time management disposition	3.39	0.55	0.04	-0.01	0.02	—			
5. Life satisfaction	5.35	0.76	0.02	-0.08	-0.10^**^	0.29^**^	—		
6. Core self-evaluations	3.66	0.65	0.01	0.04	-0.02	0.28^**^	0.58^**^	—	
7. Mental health	0.70	0.15	0.01	-0.03	-0.07	0.22^**^	0.54^**^	0.59^**^	—

*Note*: Gender is a dummy variable, male = 0, female = 1; *N* = 398; ^*^p-value < 0.05, ^**^p-value < 0.01, and ^***^p-value < 0.001, the same as below

### Mediated modeling testing

Using the hierarchical regression analysis method proposed by Wen et al. [45], all variables were standardized, and a regression analysis of mental health was conducted on time management disposition and on core self-evaluation. With time management disposition in the first tier and life satisfaction in the second tier (see Table 2), life satisfaction had a significant predictive effect on mental health after it was added to the model as a mediating variable (β = 0.52, *p* < 0.001), but time management disposition had no significant predictive effect on mental health (β = 0.07, *p* = 0.084). This indicates that life satisfaction plays a complete mediating role between time management disposition and mental health. The total effect of time management disposition on mental health was 0.22, the direct effect was 0.07, the mediation effect was 0.15, the confidence interval was [0.09, 0.22], and the pooled proportion of the total effect was 0.682. Therefore, 68.2% of the effect of time management disposition on mental health occurred through life satisfaction, supporting *H*_1_.

**Table 2:** Regression analysis of mental health on time management disposition and core self-evaluation

Predictive variables	Mental health			
	*R*	*R* ^2^	*F*	β
Level 1 Time management disposition	0.22	0.05	23.16^***^	0.22^***^
Level 2 Time management disposition	0.54	0.29	94.28^***^	0.07
Life satisfaction				0.52^***^

Thereafter, model 59 (a moderated mediation model) was used in the SPSS 23.0 macro program PROCESS to analyze whether core self-evaluations moderate the relationship between time management disposition, life satisfaction, and mental health. If the 95% confidence interval does not contain 0, the mediation effect holds [38]. As shown in Table 3, time management disposition significantly predicted life satisfaction (β = 0.14, *p* < 0.001), whereas the interaction between time management disposition and core self-evaluations did not significantly predict life satisfaction (β=-0.01, *p* = 0.671). Therefore, the first half of the mediating effect of time management disposition on mental health was not significantly moderated by core self-evaluation. Life satisfaction significantly predicted mental health (β = 0.28, *p* < 0.001), and the interaction between life satisfaction and core self-evaluations significantly predicted mental health (β=-0.11, *p* < 0.001). Therefore, the second half of the mediating effect of time management disposition on mental health was moderated by core self-evaluation. Thus, Hypothesis *H*_2*c*_ was validated.

**Table 3:** Mediated modeling testing of time management disposition on mental health

Regression equations		Overall fit index				Significance of Regression coefficients	
Outcome variables	Predictive variables	*R*	*R* ^*2*^	*R* ^*2*^	β	95% CI	*t*
	Time management disposition				0.14	[0.06, 0.22]	3.46^***^
Life satisfaction	Core self-evaluations	0.59	0.35	80.30^***^	0.54	[0.46, 0.62]	13.54^***^
	Time management disposition×Core self-evaluations				-0.01	[-0.08, 0.05]	-0.42
	Time management disposition				0.03	[-0.04, 0.11]	-0.85
Mental health	Life satisfaction	0.65	0.42	81.87	0.28	[0.19, 0.37]	-6.29^***^
	Core self-evaluations				0.41	[0.32, 0.50]	-9.21^***^
	Life satisfaction×Core self-evaluations				-0.11	[-0.16, -0.05]	3.51^***^

Further, Model 14 in PROCESS was used to test whether the size of the mediating effect varied with the change in the moderator variable. The results showed that the mediating effect of time management disposition on mental health was significant at two levels: between the core self-evaluation below one standard deviation and the mean one below standard deviation (see Table 4).

**Table 4:** The mediating effect of different core self-evaluations on time management disposition affecting mental health through core self-evaluations

Core self-evaluations	Effect size	Bootstrap *SE*	95% CI
Below M–1 *SD*	0.11	0.03	[0.06, 0.17]
Between M + 1 *SD*	0.08	0.02	[0.05, 0.13]
Above M + 1 *SD*	0.05	0.02	[0.02, 0.09]

**Fig. 2:**
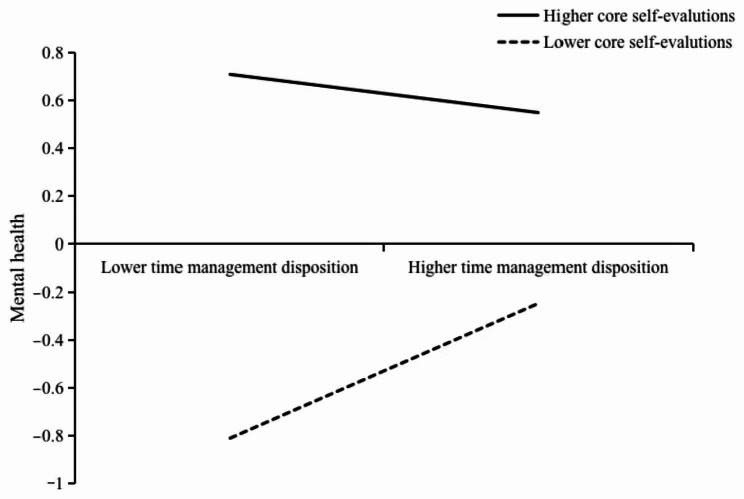
The moderating effect of core self-evaluations on the relationship between time management disposition and mental health

**Fig. 3:**
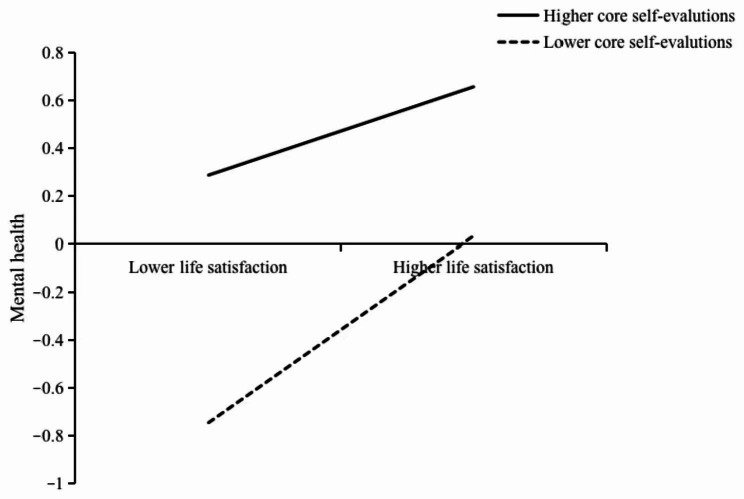
The moderating effect of core self-evaluations on the relationship between life satisfaction and mental health

Using one standard deviation of the mean of high and low core self-evaluations as the standard, the high core self-evaluation group (*M* + 1*SD*) and the low core self-evaluation groups (*M*-1*SD*) were distinguished, and a simple slope analysis was performed (see Figs. 2 and 3). Figure 2 shows that, for individuals with lower core self-evaluations, time management disposition positively and significantly predicted mental health (B_simple_=0.28, *t* = 4.97, 95% CI=[0.17, 0.39], *p* < 0.001). For individuals with higher core self-evaluations, time management disposition did not significantly predict mental health (B_simple_=-0.08, *t*=-1.72, 95% CI=[-0.17, 0.01], *p* = 0.087). Figure 3 shows that for individuals with lower core self-evaluations, life satisfaction positively and significantly predicted mental health (B_simple_=0.39, *t* = 7.63, 95% CI=[0.29, 0.49], *p* < 0.001). For individuals with higher core self-evaluations, life satisfaction was less positively predictive of mental health (B_simple_=-0.08, *t*=-1.72, 95% CI=[-0.17, 0.01], *p* = 0.087).

## Discussion

The findings of current study help to clarify how and under what conditions there is a significant relationship between postgraduate time management tendencies and mental health. Further, they have certain theoretical significance for deepening and expanding the research on the relationship between postgraduate time management disposition and mental health, and provide a practical reference for the prevention and intervention of mental health problems in postgraduate groups.

The current study found a significant positive correlation between time management disposition and mental health; that is, the stronger the time management disposition, the less likely the occurrence of psychological problems, and the higher the mental health level. This is consistent with existing research findings [46]. Individuals with higher time management disposition tend to have stronger time management ability, and this ability will positively and subtly affect the individuals’ mental health. In addition to completing regular academic tasks, graduate students may also undertake various complex and challenging scientific research tasks, and time is undoubtedly a valuable resource. Having strong time management ability will help the graduate students to improve their work and study efficiency, and thus develop a stronger sense of control over their lives and have fewer psychological problems even under high pressure from studies and research. This finding supports the developmental model of “positive traits → positive factors” developed in the current study. From the perspective of positive psychology, high core self-evaluation, as a positive inner cognition, is an important resource for individual physical and mental health and development and should be valued [47].

In addition, life satisfaction played a mediating role between time management disposition and mental health, and individuals with stronger time management tendencies had higher life satisfaction and fewer psychological problems. These findings are also consistent with a previous study [48]. Individuals with strong time management disposition are good at scientific management and rational control of time, which helps them to experience more positive emotions in life, thereby improving life satisfaction. Life satisfaction, as a subjective experience, is also closely related to mental health [49, 50]. When graduate students have a high life satisfaction level, their physical and mental health is maintained at a good level. This finding shows that in the development model of “positive traits→positive factors”, an individual’s positive traits may also affect another positive factor through one positive factor to achieve good and sustainable physical and mental development. Therefore, school educators can attempt to guide and train postgraduates on time management ability and thus improve their ability to manage their studies and life, thereby improving their life satisfaction as well as physical and mental health.

The current study verified the moderating role of core self-evaluation as an important protective factor for mental health. First, core self-evaluation moderates the direct path of “time management disposition → mental health”. Time management disposition significantly and positively predicted the mental health of individuals with low core self-evaluation but the predictive effect was not significant for those with high self-evaluation. This may be because, for individuals with low core self-evaluation, improving time management ability will reduce the sense of stress due to lack of time to a certain extent [26], thereby reducing the possibility of psychological problems. Second, core self-evaluation moderated the second half of the mediating effect of “time management tendency→life satisfaction→mental health.” For individuals with high core self-evaluation levels, the negative effect of life satisfaction on mental health scores was weakened. In general, the moderating effect of core self-evaluation is shown as a relieving effect, and high core self-evaluation can effectively maintain a good level of individual mental health and reduce the possibility of psychological problems. This is consistent with existing research findings that core self-evaluation can moderate the impact of related factors on mental health [51]. To a certain extent, this is also consistent with the assumptions of the “protection factor-protection factor” model [25]. That is, core self-evaluation and life satisfaction are protective factors that can jointly maintain and promote mental health. Finally, the current study did not find a moderating effect of core self-evaluation on the first half of the mediating effect of “time management tendency → life satisfaction → mental health.” This indicates that time management tendency significantly predicts life satisfaction for individuals with both high and low core self-evaluations [20], making the difference between the two insignificant.

This may be because graduate students are in a special stage of transition from early to mid-adulthood, have a longer moratorium than undergraduates do, and have a higher education level. Their beliefs and expectations regarding how they plan and use their time largely determine their happiness [46]. This in turn affects their life satisfaction, which is less affected by the level of core self-evaluation in the process. Therefore, postgraduate tutors and school educators can take up relevant measures, such as specialized mental training and positive affirmation of postgraduates’ personal achievements, to improve the core self-evaluation level of postgraduate groups and thus protect and improve their mental health.

The current study’s theoretical contribution includes improving postgraduates’ mental health by constructing a developmental model of “positive traits→positive factors.” From a development perspective, it explores the practical method, takes service guidance orientation as the core, discusses the relationship between postgraduate time management tendency and mental health, reveals the mediating role of life satisfaction and the moderating role of core self-evaluation, and deepens the research on postgraduate time management. Understanding the impact mechanism of mental health has a certain theoretical value. Practically, the current study focused on finding that preventing and intervening in postgraduate mental health is crucial. The mediating effect of life satisfaction suggests that postgraduate tutors and school educators can improve postgraduates’ ability to manage their studies and life through training, thereby improving their life satisfaction and mental health. The moderating role of core self-evaluation indicates that positive core self-evaluation, as an important protective factor for graduate students’ mental health, should be highly valued.

However, the current study has some limitations that need to be further improved and explored. First, the data in the current study are cross-sectional; therefore, it is difficult to determine the causal relationship between the variables. Subsequent studies could consider combining longitudinal tracking methods to test the findings of the current study. Second, the current study has not examined postgraduate academic stress as a factor that may affect the mental health of postgraduates to a certain extent. Subsequent research can consider controlling for this factor and other related factors to further test the findings of the current study.

## Conclusions

The current study concludes that time management disposition is an important predictor of mental health, and life satisfaction plays a mediating role between time management disposition and mental health. Core self-evaluations played a moderating role in the second half of the direct effect of time management disposition on mental health and the mediating effect of time management disposition on mental health through life satisfaction. School educators can improve their mental health by guiding graduate students in developing good time management disposition and enhancing their life satisfaction and core self-evaluation levels.

## Data Availability

Data and materials are available on request from the corresponding author.
